# Comparison of volumetric and functional parameters in simultaneous cardiac PET/MR: feasibility of volumetric assessment with residual activity from prior PET/CT

**DOI:** 10.1007/s00330-017-4896-7

**Published:** 2017-06-19

**Authors:** C. Lücke, B. Oppolzer, P. Werner, B. Foldyna, P. Lurz, T. Jochimsen, B. Brenneis, L. Lehmkuhl, B. Sattler, M. Grothoff, H. Barthel, O. Sabri, M. Gutberlet

**Affiliations:** 1 Department of Diagnostic and Interventional Radiology, University Leipzig – Heart Center, Strümpellstr. 39, 04289 Leipzig, Germany; 2 Department of Nuclear Medicine, University Hospital Leipzig, Leipzig, Germany; 3 Cardiac MR PET CT Program, Massachusetts General Hospital - Harvard Medical School, Boston, MA USA; 4 Clinic for Internal Medicine/Cardiology, University Leipzig – Heart Center, Leipzig, Germany; 5 Radiologische Klinik, Herz- und Gefäß-Klinik GmbH, Bad Neustadt, Germany

**Keywords:** Positron emission tomography/methods*, Magnetic resonance imaging/methods*, Cardiac-gated imaging techniques, Cardiac volume, Cardiac function test

## Abstract

**Objective:**

To compare cardiac left ventricular (LV) parameters in simultaneously acquired hybrid fluorine-18-fluorodeoxyglucose ([18F] FDG) positron emission tomography/magnetic resonance imaging (PET/MRI) in patients with residual tracer activity of upstream PET/CT.

**Methods:**

Twenty-nine patients (23 men, age 58±17 years) underwent cardiac PET/MRI either directly after a non-cardiac PET/CT with homogenous cardiac [18F] FDG uptake (n=20) or for viability assessment (n=9). Gated cardiac [18F] FDG PET and cine MR sequences were acquired simultaneously and evaluated blinded to the cross-imaging results. Image quality (IQ), end-diastolic (LVEDV), end-systolic volume (LVESV), ejection fraction (LVEF) and myocardial mass (LVMM) were measured. Pearson correlation and intraclass correlation coefficient (ICC), regression and a Bland–Altman analysis were assessed.

**Results:**

Except LVMM, volumetric and functional LV parameters demonstrated high correlations (LVESV: r=0.97, LVEDV: r=0.95, LVEF: r=0.91, LVMM: r=0.87, each *p*<0.05), but wide limits of agreement (LOA) for LVEDV (−25.3–82.5ml); LVESV (−33.1–72.7ml); LVEF (−18.9–14.8%) and LVMM (−78.2–43.2g). Intra- and interobserver reliability were very high (ICC≥0.95) for all parameters, except for MR-LVEF (ICC=0.87). PET-IQ (0–3) was high (mean: 2.2±0.9) with significant influence on LVMM calculations only.

**Conclusion:**

In simultaneously acquired cardiac PET/MRI data, LVEDV, LVESV and LVEF show good agreement. However, the agreement seems to be limited if cardiac PET/MRI follows PET/CT and only the residual activity is used.

***Key Points*:**

• *[* ^*18*^ *F] FDG PET-MRI is feasible with residual [* ^*18*^ *F] FDG activity in patients with homogenous cardiac uptake.*

*• Cardiac volumes and function assessed by PET/MRI show good agreement.*

*• LVEDV and LVESV are underestimated; PET overestimates LVMM and LVEF.*

*• Cardiac PET and MRI data correlate better when acquired simultaneously than sequentially.*

*• PET and MRI should not assess LV parameters interchangeably.*

## Introduction

Cardiac magnetic resonance imaging (MRI) provides incremental impact on clinical decision-making by delivering data about myocardial anatomy, function, tissue characterization, perfusion, diffusion and also metabolism when using MR spectroscopy [1]. MRI is the clinical standard of reference for the volumetric and functional assessment of the heart [2–5]. Cardiac positron emission tomography (PET) can provide precise information on cardiac perfusion, viability, myocardial metabolism and other molecular processes [6]. [^18^F] FDG cardiac PET visualizes the glucose metabolism in the viable myocyte and is the gold standard for the direct visualization of viability [7]. The unique combination of PET and MRI offers a comprehensive approach for cardiac diseases [6, 8], i.e. in inflammatory diseases like myocarditis, sarcoidosis or rejection after heart transplantation [9, 10]. Both modalities can assess cardiac function.

Recently, hybrid PET/MR systems have been introduced [11], and allow the evaluation of simultaneously acquired volumetric cardiac data of both modalities. Thus, the question arises whether volumetric parameters could be used interchangeably. Validation studies on co-registering of sequentially acquired [^18^F] FDG-PET and MRI data sets showed a good agreement of LV parameters with a systematic bias [12–14]. However, inter- and/or intraday variability of LV parameters can occur, if not acquired simultaneously [14]. Even the inherent differences in acquisition times between PET and MRI can cause misalignment, mostly due to patient motion or respiration [8].

LV function and especially the normalized LVESV (LVESVI) is a relevant prognostic factor in patients with coronary artery disease (CAD) and a LVESVI > 100 ml/m^2^ predicts worse outcome [15]. In comparative studies, [^18^F] FDG-PET showed a non-significant tendency to overestimate LVESV and to underestimate LVEDV, resulting in an underestimation of LVEF values [2]. The lower temporal and spatial resolution of cardiac [^18^F] FDG-PET [2] as compared to MRI [8] might be a reason. But, the temporal resolution of gated [^18^F] FDG-PET has been improved lately and spatial resolution of MRI is lower in the through-plane direction. Also, cardiac function by [^18^F] FDG-PET has been mainly evaluated in patients with CAD, whom often show a heterogeneous myocardial uptake, while patients who undergo [^18^F] FDG-PET for non-cardiac reasons sometimes show a homogenous uptake [16].

The aims of this study were:

1. To test whether an observed homogeneous uptake is sufficient to perform a simultaneous cardiac PET/MR with the residual activity after a PET/CT for non-cardiac indication.
2. To analyse whether the acquired data is sufficient for a LV volumetric and functional analysis.
3. To assess whether bias and limits of agreement are small enough in a way that cardiac PET or MRI volumetric and functional data could be used interchangeably.

## Materials and methods

### Patients

The present study was performed under the guidelines of the 1964 Helsinki Declaration and approved by the local ethics committee. All patients gave written informed consent and agreed to the anonymous evaluation of their data. The nine patients with CAD gave written consent for viability assessment by PET/MRI the day before. Patients who were scheduled for a non-cardiac PET/CT were asked about their willingness to participate in the PET/MR study prior to the PET/CT acquisition. Twenty of all screened patients between November 2011 and September 2015 with a sufficient, homogeneous myocardial FDG uptake in PET/CT received an additional PET/MRI. Image quality (IQ) was visually graded by the homogeneity of myocardial tracer uptake on a four-point analogue scale (3 = very homogenous, 0 = very low or heterogeneous).

Indications for PET/CT are given in Table 1. Inclusion criteria for this group were: cardiac [^18^F] FDG uptake evaluated as >1 as seen in PET/CT, and age > 18. An incomplete PET or MRI scan, contraindications for MRI, claustrophobia and state of pregnancy were exclusion criteria. Patients prior to PET/CT received the appropriate fasting protocol [17] and no additional [^18^F] FDG activity for the PET/MR examination; patients for viability assessment received a glucose load protocol [18]—modified in diabetes mellitus [19].

**Table 1:** Patient characteristics

	Total (n = 29)	No-CAD (n = 20)	CAD (n = 9)	Difference No-CAD/CAD
Male	23	15	8	*p* < 0.05
Age [years]	58 ±17	53 ±17.2	70 ±8.2	*p* < 0.05
PET/MR start [hours after injection]	2.3 ±1.1	2.8 ±0.9	1.1 ±0.1	*p* < 0.0001
PET image quality (IQ): mean of homogeneity (visual analogue scale *)	2.2 ±0.9	2.5 ±0.7	1.6 ±1.1	n.s.
Mean activity injected [MBq]	330.7 ±61	336.0 ±61	318.0 ±64	n.s.
CAD type				
3-vessel disease	5	0	5	
2-vessel disease	4	0	4	
1-vessel disease	0	0	0	
Diabetes mellitus	2	-	2	
Clinical indication for [^18^F] FDG PET/CT scan				
Testicular cancer	4	4	-	
Lymphoma	3	3	-	
ENT tumour	3	3	-	
Lung cancer	3	3	-	
Bone/soft-tissue tumour	2	2	-	
Breast cancer	1	1	-	
Melanoma	1	1	-	
Fever of unknown origin	4	4	-	

The patient characteristics, including type of coronary artery disease (CAD), age, gender, hours after injection of [^18^F] FDG, injected activity and the homogeneity of uptake and indication for PET-CT are given in the table. *Homogeneity of myocardial tracer uptake was visually graded on a four-point analogue scale (3 = very homogenous uptake, 0 = very low or heterogeneous uptake)

### PET/MR protocol

Patients were examined in supine position using a simultaneous hybrid PET/MR system (mMR Biograph; Siemens, Erlangen, Germany) with dedicated phased-array surface coils (*mMRBody, mMRSpine*) for combined PET/MR measurements [3]. For attenuation correction (AC) of the PET data, a two-point Dixon volumetric interpolated breath-hold examination (Dixon-VIBE) sequence was acquired in end-expiration [20, 21]. The cardiac MRI and PET emission data were acquired simultaneously. The PET data was acquired in list mode. The MRI protocol consisted of a single-slice, steady-state free precession (SSFP) sequence acquired in a two-chamber and a four-chamber view and a stack of contiguous short axis slices. Short-axis SSFP sequences covered the whole heart during several end-expiration breath holds. The repetition time (TR) was automatically adjusted according to the volunteer /patient´s heart rate. The following parameters were applied: slice thickness/increment: 8/8 mm, echo time (TE): 1.51 ms, a mean heart rate-dependent repetition time (TR): 50 ms, flip angle: 50°, typical field of view (FoV): 292.5 x 360 mm, matrix: 208 x 256 voxels (voxel size: 1.4 × 1.4 × 8.0 mm^3^), retrospectively gated with a fixed number of 25 reconstructed phases. From the list-mode PET data, a fixed number of 16 gates were reconstructed using the ordered subset expectation maximization algorithm (3 iterations, 21 subsets, 3-mm gauss filter). The resulting matrix consisted of 128 x 128 voxels (voxel size: 1.4 × 1.4 × 2.03 mm^3^).

### Data analysis

Three observers evaluated the datasets. Observer (O1) evaluated the MRI and PET datasets. Observer 2 (O2) evaluated only PET and observer 3 (O3) evaluated only MRI data, all blinded to the results of the other method. To assess intra-observer reliability, each evaluation was repeated after a 3-month waiting period, to exclude memory bias. The first measurement is named M1, the second M2 and for the first observer with the modality as index (e.g. M1_PET_ or M1_MRI_).

### MRI data analysis

**Fig. 1:**
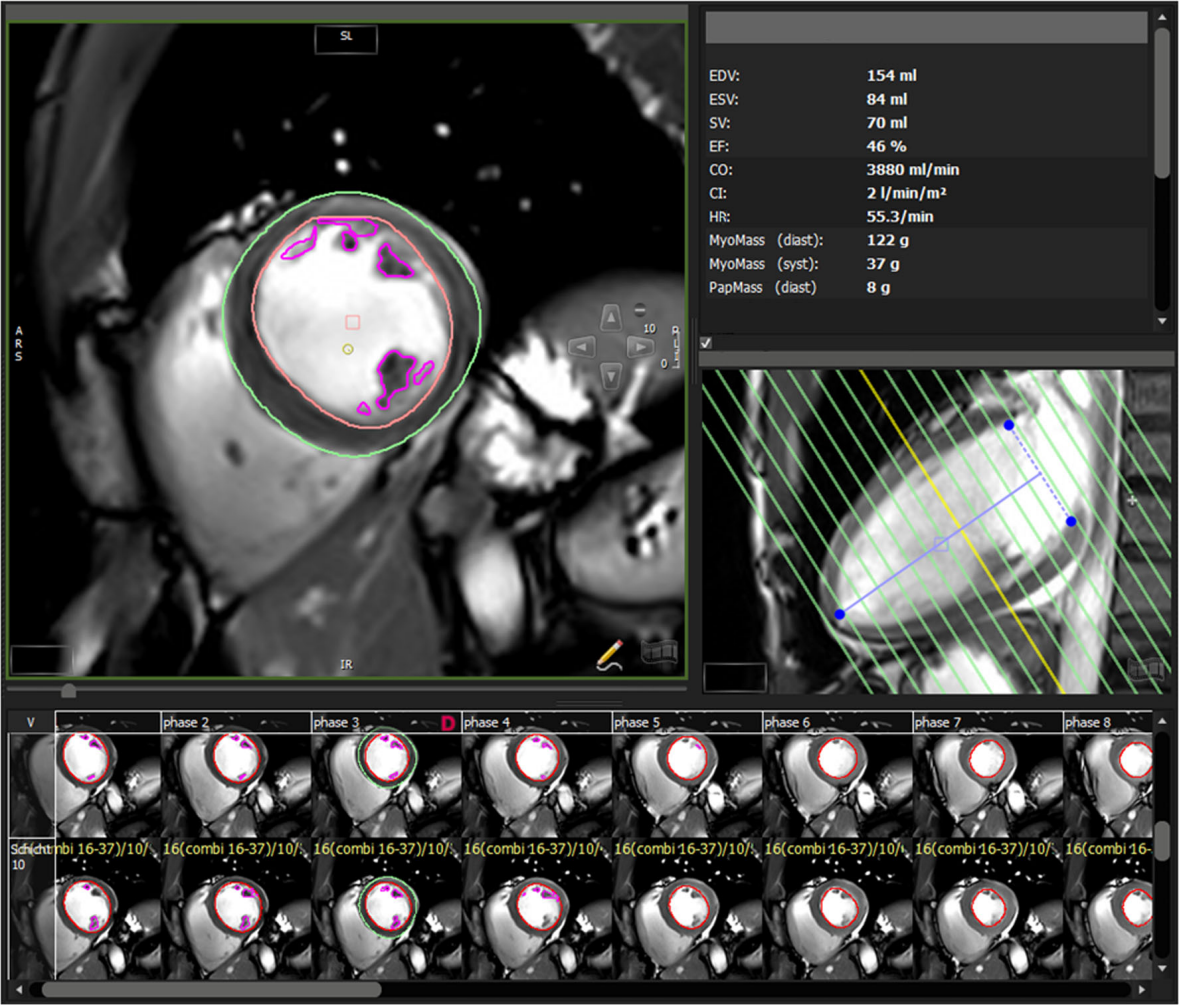
Evaluation of short axis cine MR images using cmr42. *MRI data evaluation of a simultaneously acquired PET/MR data set in a patient with a previous non-cardiac PET/CT examination:* Delineation of the epicardial and endocardial contours in the short axis orientation in the end-diastole (phase 3) is shown in the left upper frame. The image locator in the two-chamber view is depicted in the left mid-centre. The volumes of the ventricle and the calculated ejection fraction are depicted on the top right centre of the image. Note that papillary muscles were determined separately (*violet segmentation*)

The MRI data was evaluated with CMR42 [Version 4.0.2 (151), Circle Cardiovascular Imaging Inc., Calgary, Canada]. The observer was blinded to the results of the gated [^18^F] FDG-PET analysis. LVEDV, LVESV and LVMM were assessed using Simpson´s method. Epicardial and papillary contours were drawn only in end-diastole and endocardial contours semi-automatically in all cardiac phases (Fig. 1). Total LVMM was calculated as the sum of the mass of the papillary muscles and the compacted myocardial mass, and LVEF was calculated with the usual formula [22]: (LVEDV-LVESV)/LVEDV.

### PET data analysis

**Fig. 2:**
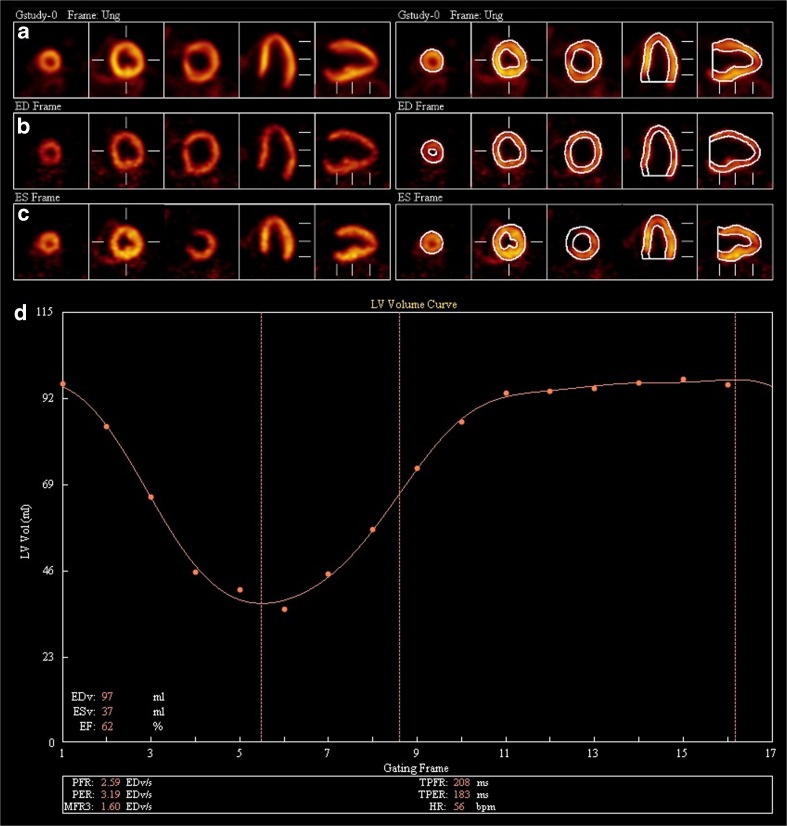
Evaluation of gated PET data using Corridor4DM. *Cardiac PET images of the LV of a patient after a previous PET/CT. Even with fasting using the residual FDG uptake, the image quality was scored 3 (very homogenous myocardial FDG uptake):* Summed frames (A), end-diastolic frames (B), end-systolic frames (C) in three short axes, horizontal long axis and vertical long axis views are shown. The delineation of the left ventricular borders is processed automatically from the PET images (*top right image*) delineation. The time-volume curve of the left ventricle over the different cardiac phases is shown graphically together with the volumetric results in (D). Note that anatomical structures such as the papillary muscles can be depicted better during systole (B) than during diastole (B)

The co-registration of the averaged PET and Dixon-VIBE data—used for AC—was visually inspected to rule out misclassifications prior to the analysis with Corridor 4DM (Ann Arbor, MI, USA, Version 6.1.5) [22]. The mitral valve and apex were visually identified, tagged manually and an initial estimate of the ventricles were created by the software on the basis of a two-dimensional gradient image [2]. LVEDV, LVESV, LVEF and LVMM were calculated automatically from the computed epi- and endocardial contours [23] (Fig. 2).

### Statistical analysis

Statistical analyses were performed using MedCalc for Windows, Vers.12.5 (MedCalc Software, Ostend, Belgium). Mean differences between both modalities were tested using the Student’s *t* test for paired samples. A *p* value < 0.05 was considered to be statistically significant. Correlation between methods was calculated using Pearson coefficients.

Intra- and interobserver reliability were assessed as intraclass correlation coefficient (ICC) and analysed as follows:

- Intra-observer reliability:
- M1 and M2 of O2 and O3, respectively.
- Inter-observer reliability:
- M1_PET_ of O1 and M1 of O2;
- M1_MRI_ of O1 and M1 of O3
- Inter-modality reliability:
- M1 of O2 and M1 of O3.

Furthermore, linear regression analysis was performed and Bland–Altman plots [24] generated.

## Results

### Patient characteristics

Twenty-nine patients (23 male, 6 female) were included—detailed characteristics are shown in Table 1. Twenty patients underwent a non-cardiac PET/CT, and the other nine patients with CAD received viability assessment. Mean study population age was 58 ±17 years, while the CAD patients were significantly older. Patients who underwent PET/MR after PET/CT had no history of cardiac diseases (non-CAD).

### Image quality

All patients showed a diagnostic IQ; non-CAD and CAD patients had a mean IQ of 2.5 ±0.7 and 1.6 ±1.1, respectively (*p* < 0.0001). The IQ was ≤1 in six out of nine CAD patients and >1 in all non-CAD patients. Two CAD patients had diabetes mellitus without influencing the IQ of the FDG-PET. Nevertheless, the IQ influenced negatively the correlation of LVMM (Table 2).

**Table 2:** Influence of PET image quality (IQ) on Pearson correlation (r) between PET and MR assessment of LV parameters

Image quality (IQ)	LVEDV	*p* value	LVESV	*p* value	LVEF	*p* value	LVMM	*p* value
0–1 [n = 6] only CAD patients	0.96	<0.05	0.99	<0.01	0.98	<0.05	0.79	0.2
2 [n = 9]	0.98	<0.0001	0.97	<0.0001	0.84	<0.01	0.90	<0.01
3 [n = 14]	0.94	<0.0001	0.97	<0.0001	0.89	<0.0001	0.85	<0.0001

### Acquisition time, heart rate (HR) and radiation exposure

Acquisition time was 900 ±0 s, i.e. 15 min for gated PET and 229 ±57 s (i.e. 3.82 min) for MRI. The mean HR during the MR acquisition was 64 bpm (SD: ±13; range: 41–94 bpm), and the resulting mean RR interval was 970 ms (SD: ±196; range: 640–1475 ms). As expected, the mean HR and other HR-dependent parameters of the PET acquisition were comparable to the data of the simultaneously acquired MR acquisition with a mean PET HR of 65 bpm (SD: ±13; range: 48–93 bpm), and a resulting mean RR interval of 950 ms (SD: ±173; range: 647–1237 ms).

Initially administered activity for the PET/CT was 330 ±61 MBq resulting in an effective dose of 6.3 ±1.2 mSv. Due to PET/CT being prior to PET/MR in the majority of patients, PET/MR was performed 2.3 ±1.1h after the administration of [^18^F] FDG intravenously. It took approximately 30 min to reconstruct the gated PET from the list-mode raw data. Static PET could be fused with static MR images with dedicated PET/MR software (syngo.via VA20, SIEMENS Healthcare) for clinical reporting.

### LV volumetric and functional parameters

**Fig. 3:**
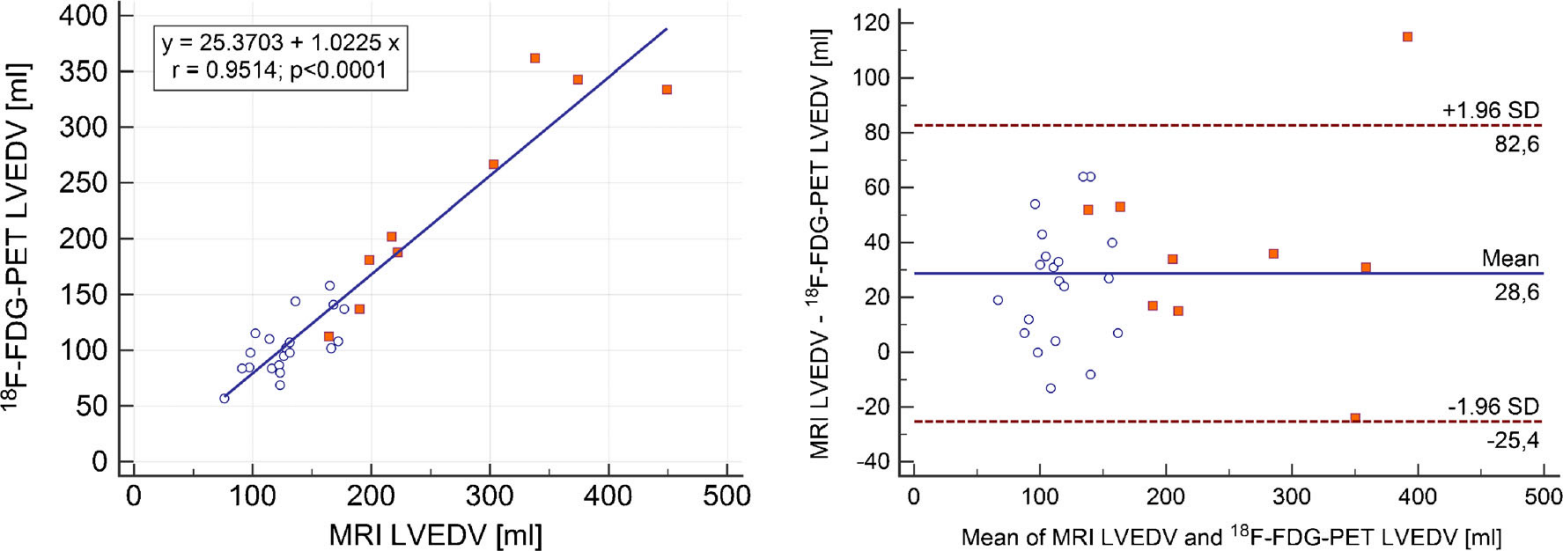
Comparison of simultaneously acquired [^18^F] FDG PET and MRI measurements of LVEDV:. (A) Regression analysis between left ventricular end-diastolic volume (LVEDV) assessed by MRI and gated PET. (B) Bland–Altman plot showing an underestimation of 28.6 ml by PET compared to MRI. Limits of agreement were −25.4 and 82.6 ml. *White dots* represent patients after PET/CT, *red dots* those with primary PET/MR for viability assessment

**Fig. 4:**
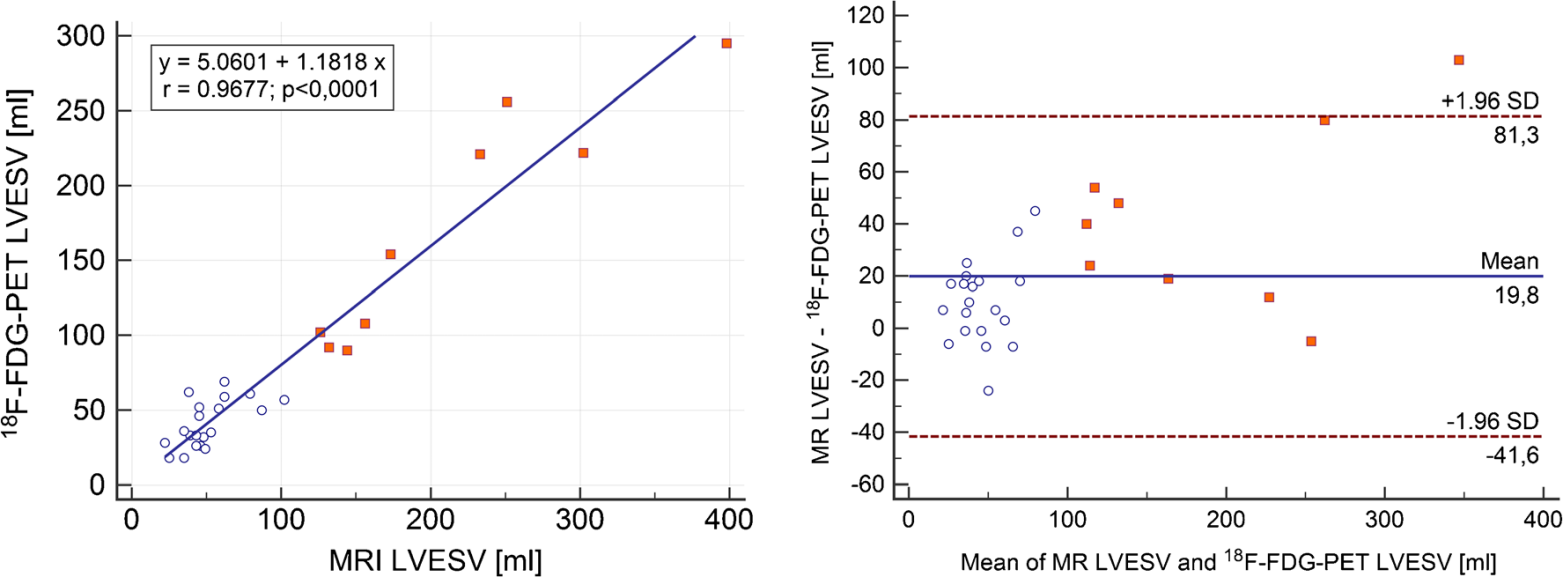
Comparison of simultaneously acquired [^18^F] FDG PET and MRI measurements of LVESV. (A) Regression analysis for LVESV assessed by MRI and gated PET (B) Bland–Altman plot with a significant underestimation (19.8 ml) by PET. Limits of agreement were −41.6 and 81.3. *White dots* represent patients after PET/CT, *red dots* primary PET/MR for viability assessment

**Fig. 5:**
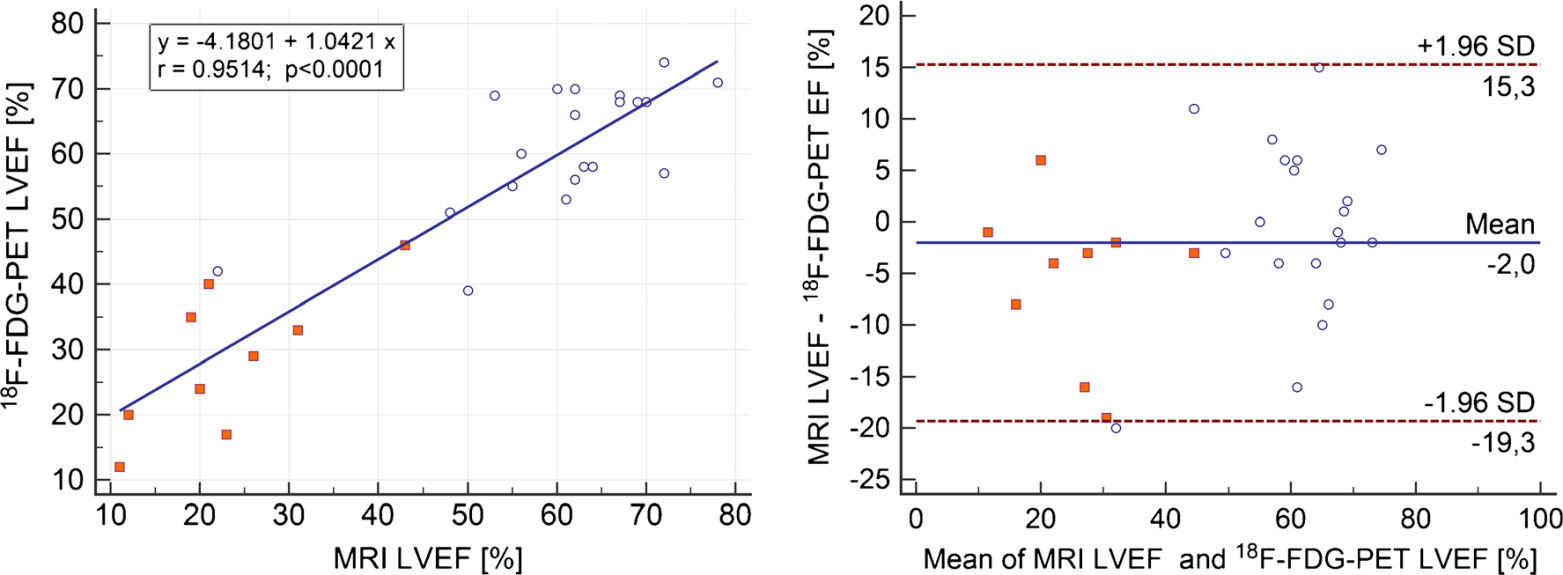
Comparison of simultaneously acquired [^18^F] FDG PET and MRI calculations of LVEF. (A) Regression analysis for LVEF fraction assessed by MRI and gated PET. (B) Bland–Altman plot with a bias of −2%, limits of agreement were −19.3% and 15.3%. *White dots* represent patients after PET/CT, *red dots* primary PET/MR for viability assessment

**Fig. 6:**
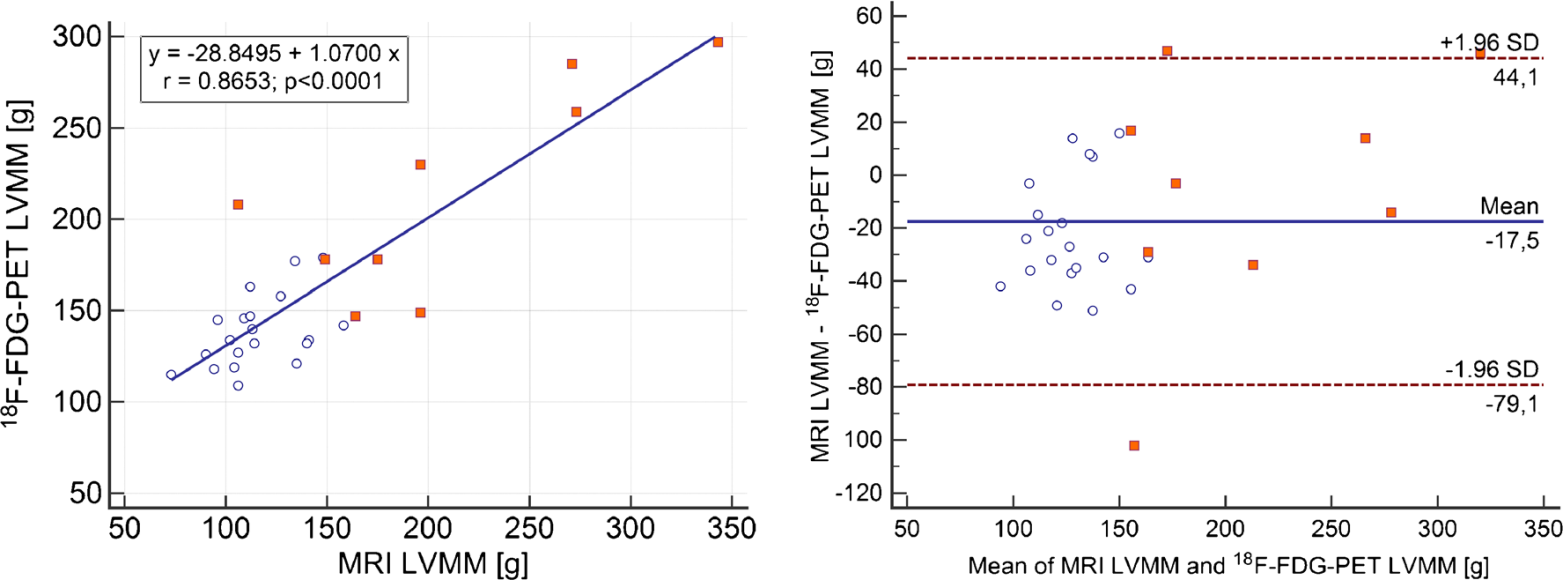
Comparison of simultaneously acquired [^18^F] FDG PET and MRI calculations of LVMM. (A) Regression analysis between left ventricular myocardial mass assessed by MRI and gated PET. (B) Bland–Altman plot shows a LVMM bias of −17.5 g. LOA values were – 79.1 and 44.1. *White dots* represent patients after PET/CT, *red dots* primary PET/MR for viability assessment

A summary of the results is shown in Table 3. The mean LVEDV measured by MRI was 173.0 ±89.1 mL, and by PET 144.4 ±82.9 mL (*p* < 0.0001) with a correlation of r = 0.95 (*p* < 0.0001) and a regression equation y = 25.3703 + 1.0225x (R^2^ = 0.91). Bland–Altman analysis revealed an underestimated LVEDV by PET (28.6 ±28 mL; Fig. 3). The mean LVESV measured by MRI was 101.1 ±92.2 mL, and by PET 81.2 ±75.5 mL (*p* = 0.0005) with a correlation of r = 0.97 (*p* = 0.0001) and a regression equation y = 5.0601 + 1.1818x (R^2^ = 0.94). Bland–Altman analysis revealed an underestimated LVESV by PET (19.0 ±26.9 mL; Fig. 4). Mean LVEF measured by MRI was 48.9 ±21.0%, and by PET 51.0 ±18.4% (n.s.) with a correlation of r = 0.91 (*p* < 0.0001) and regression equation y = −4.1801 + 1.0421x (R^2^ = 0.83). Bland–Altman analysis revealed only a small bias of −2.0 ±8.6% (Fig. 5). Mean LVMM measured by MRI was 144.4 ±61.4 g, and by PET 161.9 ±49.6 g with a correlation of r = 0.87 (*p* < 0.0001) and regression equation y = −28.8795 + 1.0700x (R^2^ = 0.75). Bland–Altman analysis revealed an overestimated LVMM by PET (−17.5 ±31.0 g; Fig. 6).

**Table 3:** Volumetric and functional results of MRI and cardiac PET in comparison to sequential MRI and PET trials in the literature

		MRI (mean, SD)	PET (mean, SD)	Correlation	Bias	Upper LOA	Lower LOA
Current study [n = 29]	LVEDV [ml]	173 ±89	144 ±82	r = 0.95; *p* < 0.0001	28.6	82.5	−25.3
Subgroup CAD [n = 9] No-CAD [n = 20]		273 ±98 128 ±29	236 ±93 103 ±26	r = 0.92 r = 0.69	36.6 25.1	109.9 68.0	−17.9 −19.9
Khorsand et al. [12] [n = 20]		189 ±99	170 ±68	r = 0.92; *p* < 0.0001	−19	71.2	−109.2
Schäfer et al. [2] [n = 42]		176 ±53	177 ±56	r = 0.94; *p* < 0.00001	0	39	−39
Slart et al. [11] [n = 38]		131 ±57	117 ±56	r = 0.91; *p* < 0.001	19.6	56.3	−17.1
Current study [n = 29]	LVESV [ml]	101 ±92	81 ±76	r = 0.97; *p* < 0.0001	19.8	72.7	−33.1
Subgroup CAD [n = 9] No-CAD [n = 20]		213 ±92 51 ±20	171 ±79 41 ±16	r = 0.93 r = 0.62	41.7 10.0	108.5 41.3	−25.2 −21.3
Khorsand et al. [12] [n = 20]		112 ±93	101 ±60	r = 0.93; *p* < 0.0001	−11	75.2	−97.2
Schäfer et al. [2] [n = 42]		118 ±50	126 ±52	r = 0.95; *p* < 0.00001	−7	24	−38
Slart et al. [11] [n = 38]		91 ±12	85 ±51	r = 0.94; *p* < 0.0001	12.5	39.0	−14.0
Current study [n = 29]	LVEF [%]	49 ±21	51 ±18	r = 0.91; *p* < 0.0001	−2	14.8	−18.9
Subgroup CAD [n = 9] No-CAD [n = 20]		23 ±9.8 61 ±12	28.5 ±11 61 ±10	r = 0.74 r = 0.70	−5.6 −0.5	9.6 16.5	−20.7 −17.4
Khorsand et al. [12] [n = 20]		46 ±18	44 ±13	r = 0.85; *p* < 0.0001	−3	16.6	−22.6
Schäfer et al. [2] [n = 42]		35 ±11	31 ±8	r = 0.94; *p* < 0.00001	4	13	−5
Slart et al. [11] [n = 38]		33 ±12	33 ±11	r = 0.96; *p* < 0.0001	3.4	7.7	−0.9
Current study [n = 29]	LVMM [g]	144 ±61	162 ±50	r = 0.87; *p* < 0.0001	−17.5	43.2	−78.2
Subgroup CAD [n = 9] No-CAD [n = 20]		208 ±74 116 ±22	215 ±57 138 ±20	r = 0.78 r = 0.50	−6.4 −22.5	84 18.2	−96.9 −63.2
Khorsand et al. [12] [n = 20]		200 ±46	196 ±44	r = 0.75; *p* < 0.001	−4	58.7	−66.7

Results are given in mean with standard deviations (SDs). Furthermore, the Pearson correlation coefficient (r), and the bias and the lower and upper limits of agreement (LOA) of the Bland–Altman analysis are given.

#### Reproducibility

Intraobserver reliability was very high and comparable in both modalities for all LV parameters (ICC ≥ 0.95). Interobserver reliability was very high for all LV parameters in PET (ICC ≥ 0.96). In MRI, the interobserver reliability was high for LVEF (ICC = 0.87), and very high for all other LV parameters (Table 4). The standard error of the estimate (SEE) of the regression analysis was 27.9 ml for EDV, 23.7 ml for ESV, 8.7% for EF and 31.3 g for LVMM.

**Table 4:** Intra- and interobserver variability: Intraclass coefficient with a 95% confidence interval (CI), bias and LOA of the Bland–Altman analysis

		MRI			PET		
		ICC [95% CI]	Bias	LOA	ICC [95% CI]	Bias	LOA
Intraobserver	LVEDV (ml)	0.99 [0.98–0.99]	−4.8	−31.5; 22.0	0.98 [0.96–0.99]	2.4	−26.5; 31.4
	LVESV (ml)	1 [0.99–1]	−2.9	−20.1; 14.2	0.99 [0.98–0.99]	−0.2	−22.3; 21.8
	LVEF (%)	0.96 [0.92–0.98]	0.5	−10.2; 11.2	0.97 [0.95–0.99]	1.4	−6.3; 9.2
	LVMM (g)	0.95 [0.89–0.97]	1.9	−35.6; 39.4	0.97 [0.94–0.99]	0.8	−21.2; 22.8
Interobserver	LVEDV (ml)	0.96 [0.92–0.98]	−3.9	−54.6; 46.8	0.99 [0.99–1]	−0.3	−18.3; 17.6
	LVESV (ml)	0.97 [0.93–0.98]	−7.9	−55.4; 39.6	0.99 [0.97–0.99]	−4.1	−28.6; 20.4
	LVEF (%)	0.87 [0.74–0.93]	4.1	−16.2; 24.4	0.96 [0.92–0.98]	2.0	−8.2; 12.1
	LVMM (g)	0.94 [0.88–0.97]	18.6	−19.5; 56.6	0.97 [0.94–0.99]	−3.3	−26.3; 19.7

## Discussion

Assessment of left ventricular volumes, function and mass data is feasible with a homogenous residual activity of a previous PET/CT examination. If acquired with simultaneous cardiac PET/MR, parameters correlated well between PET and MRI readouts with only small bias, (Table 3). However, the limits of agreement (LOAs) are rather wide, such that PET and MRI functional and volumetric data could be used interchangeably.

Intra- and interobserver reliability was slightly lower in PET as compared to MRI, although MRI is a frequently used standard of reference. These results are not surprising, because the cardiac PET algorithm for the volumetric and functional evaluation does not allow much user-software interaction. Observers can only change the LV axis, the position of the apex and the mitral valve. Epicardial and endocardial contours are detected automatically and cannot be changed manually. Papillary muscles are merged into the contours or neglected due to low uptake. With more possible user interaction, the inter- and intraobserver variability increases [25]. The contours, defined in the MRI datasets, can be modified substantially and partial volume effects of the papillary muscles and the manual correction can lead to discrepancies in the different measurements. Therefore, we conclude that the higher intra- and interobserver variability deduces itself from higher user-software interaction and can be decreased by reader consensus training [26].

We acquired the SSFP sequences in expiration, because our own unpublished experience in consensus to other studies has revealed [20, 21] that this method leads to the most accurate thoracic image fusion, due to free breathing being mainly an end-expiration phase [27]. Additional respiratory gating of the PET acquisition, usually triggered by MR navigator pulses, may improve image fusion, however, at the cost of higher acquisition time [21, 27].

The correlation between MRI and PET with corresponding coefficients for LVEDV, LVESV and LVEF is comparable to other studies [2, 12, 13], except LVMM, which was lower in one study [13]. All three studies quoted in Table 3 examined patients exclusively with coronary CAD and reduced LVEF. In our study, only 31% of patients had CAD.

Different temporal resolutions can significantly alter the correct assessment of LVEF, if the end-diastolic and end-systolic phases are not detected properly [28, 29]. In patients with severely impaired LVEF, the differences between LV volumes in each phase are smaller. Therefore, a lower temporal resolution has less influence on LVEF compared to patients with normal or slightly decreased LVEF [4].

The TR of our used SSFP sequence was automatically adjusted to HR and ranged between 46–52 ms. The mean TR of our examined cohort was 50 ms (SD: ±1.3; range: 46–52), the mean HR was 64 bpm (SD: ±13; range: 41–94 bpm), the resulting mean RR interval was 970 ms (SD: ±196; range: 640–1475 ms). Therefore, in our cohort, the mean HR was rather low, and the corresponding RR interval was high, which are rather optimal conditions to achieve a high temporal resolution. Taking this information into account, we had a mean maximum phase duration of 970/20 = 48.5 ms to catch the systolic phase appropriately within 5% of the RR interval. However, a “temporal partial volume effect” may occur with a loss of precision in the determination of systolic volumes in some patients, and thus a potential underestimation of the ejection fraction.

Due to the fact that a fixed number of 16 gates were reconstructed with the PET acquisition, the mean gate length of the PET reconstruction was 60 ms (SD: ±11; range: 40–77 ms) in our cohort. Therefore, the mean temporal resolution of the PET acquisition was obviously lower than the temporal resolution of the MR acquisition with a mean percentage of the PET gate length with the MR TR of 118% (SD: ±23; range: 79–159%).

However, the reconstructed temporal resolution of MRI in our study was nearly 40% higher with a fixed number of 25 reconstructed phases per cardiac cycle in MRI as compared to a fixed number of 16 gates in cardiac PET, compared to all other cited studies (Table 2): Khorsand et al. (MRI 12–16 phases, PET: 8 gates), Schaefer et al. (MRI: 12–16 phases, PET: 8 gates) and Slart et al. (MRI: approximately 20 phases, PET: 16 gates). This difference in temporal resolution may not have significantly contributed to the differences of LV parameters between the methods, but may have contributed to the differences compared to other studies. Slart et al. had the best correlation for LVEF with an equal temporal resolution.

While the spatial “in-plane” resolution—the voxel size was 1.4 × 1.4× 8.0 mm^3^ for MRI and 1.4 × 1.4 × 2.03 mm^3^ for PET—was the same for MRI and PET, the spatial “through-plane” resolution was higher in PET. However, since the orientation in cardiac MRI is along the cardiac axis, while in PET it is along the z-axis of the patient, the spatial resolution is not directly comparable, especially if the variable position of the heart is considered. However, the higher spatial “through-plane” resolution of PET is, in our opinion, not the main factor for the difference between the results.

Comparable to other trials, the volumetric assessment of simultaneously acquired PET and MRI data showed significantly lower mean left ventricular volumes with PET as compared to MRI, also in the subgroups with and without CAD (Table 3). Furthermore, there were tendencies towards higher calculated LVEF and LVMM values with PET.

Non-CAD patients demonstrated similar results to Schaefer et al. [2], but narrower LOA and a smaller bias for LVESV as well as LVEDV compared to other studies [12, 13] and comparable results to another. LVEF showed similar bias and wider LOA [12, 13]. However, the interobserver reliability in PET is lower than in the study of Khorsand et al. (values in brackets): −0.3 ±9 mL (5 ±16 mL) for LVEDV, 2% ±5% (1 ±5%) for LVEF and −3.3 ±12 g (24 ±17 g) for LVMM, although all values did not significantly differ from 0 [12, 13].

In contrast to Khorsand et al., in our study, gated PET overestimated the mean LVMM by 18 g as compared to MRI. Also, different definitions of the LV cavity may contribute to this: In MRI, segmentation is driven by the visible anatomical structures, while in PET, algorithms of the maximal [^18^F] FDG uptake in the myocardial wall are used to define an intramyocardial centreline from which the endo- and epicardial contours are estimated [12]. Unfortunately, the algorithms behind these volumetric calculations are not fully public yet. Morphological data from simultaneously acquired MRI could be used to further improve segmentation algorithms for gated PET.

There is a lack of consensus concerning preparation of patients undergoing cardiac [^18^F] FDG-PET [16]. The nine patients for viability assessment received a glucose load [11]. In contrast, the 20 patients that underwent PET/MR as a subsequent examination after PET/CT followed a fasting protocol that suppresses body muscle [^18^F] FDG uptake but does not prevent an incidental homogenous cardiac uptake [30]. In our case, only patients with this incidental homogenous myocardial uptake after PET/CT and who agreed to undergo another PET/MRI examination and had no contraindications to MRI were included in the study. This incidental homogenous uptake occurred in approx. 20% of the patients examined, which is in line with a prior study [31]. Better image quality of the cardiac PET in patients that underwent PET/CT for non-cardiac reasons compared to that of patients with ischaemic scar tissue is probably due to the fact that the first group had presumably no history of cardiac pathologies (Table 3, Fig. 2).

However, differences in PET IQ influenced negatively the evaluation of LV parameters for LVMM calculations only (Table 2). The question of how the PET/MRI outcome parameter agreement as obtained in this feasibility study employing [18F] FDG as a viable PET tracer would translate into simultaneous PET/MRI studies utilizing blood flow PET tracers like [13N] ammonia or [83Rb] rubidium needs to be answered by subsequent studies. This especially refers to the LV function assessment in low-EF ranges in which the limited temporal resolution by the 8-bin PET gating techniques can lead to a bias.

## Limitations

If our mean reported TR of 50 ms is considered as the “real” temporal resolution of our CINE-SSFP sequence, as some authors recommend [32], rather than the reconstructed frames/phases per heartbeat, which were fixed at 25 phases, then the underestimation of PET functional parameters is not only caused by the use of residual activity, but also intrinsically due to the MR technology used itself. Furthermore, the interpretation of our results is limited due to the small number of patients, though comparable to other studies in terms of patient number and results. We examined patients with and without CAD, different to Slart, Schaefer, and Khorsand et al., who evaluated patients with known CAD and reduced LVEF. Due to the sequential study design, the average time between administration of [^18^F] FDG and PET/MR was 2.3 h ±1.2 h, resulting in a lower count rate as compared to a standard [^18^F] FDG-PET, which is performed 40–60 min after [^18^F] FDG administration [12]. However, it has been demonstrated, that the use of half of the usual activity in PET/MR seems prudent [33] and high-definition hybrid cardiac FDG PET/MR has been shown to be diagnostic using a mean activity of only 150 ±70 MBq [34], which is approximately half of the mean dose used in our study. It is comparable to the mean activity still available after 2.8 h on average in our non-CAD patients, which corresponds to 1.5 half-lifes of [^18^F] FDG or 165 min.

## Conclusion

Despite a decent correlation between volumetric analysis of simultaneously acquired LVEF and LVMM PET data, obtained with model-based automated analysis software, comparable to sequentially acquired data in the literature, biases are observed with an expected underestimation of LVEF and LVSV. However, since the LVEF especially shows wide LOAs, the methods should not be used interchangeably. Cardiac volumetric analysis is feasible from PET data in patients who incidentally present with a homogenous cardiac uptake after previous whole-body PET/CT under fasting conditions, so that the fast, automated LV segmentation algorithms from cardiac PET analysis software can still be used reliably to allow for a fast and standardized evaluation. If residual myocardial [^18^F] FDG activity is used in PET/MR examinations, one should rely on MRI SSFP cine imaging as the gold standard. However, our results support the proposal to reduce the usual applied [^18^F] FDG activity in cardiac PET/MR for viability assessment from other groups [11, 35], which is of interest in terms of patient radiation protection.

## Abbreviations

MRI: Magnetic resonance imaging
[^18^F] FDG: Fluorine-18 fluorodeoxyglucose
PET: Positron emission tomography
PET/CT: Hybrid PET/computed tomography
PET/MR: Hybrid PET/magnetic resonance imaging
LV: Left ventricular
LVEDV: Left ventricular end-diastolic volume
LVESV: Left ventricular end-systolic volume
LVEF: Left ventricular ejection fraction
LVMM: Left ventricular end-diastolic myocardial mass
LVESVI: Body surface-normalized LVESV
AC: Attenuation correction
SSFP: Steady-state free precession
LOA: Limits of agreement
O1,O2, O3: Observer 1, 2, 3
M1, M2, M3: Measurement 1, 2, 3

## References

[1] Rajwani A, Stewart MJ, Richardson JD, Child NM, Maredia N (2016). The incremental impact of cardiac MRI on clinical decision-making. Br J Radiol.

[2] Schaefer WM, Lipke CS, Nowak B, Kaiser HJ, Reinartz P, Buecker A (2004). Validation of QGS and 4D-MSPECT for quantification of left ventricular volumes and ejection fraction from gated 18F-FDG PET: comparison with cardiac MRI. J Nucl Med : Off Publ, Soc Nucl Med.

[3] Pichler BJ, Kolb A, Nagele T, Schlemmer HP (2010). PET/MRI: paving the way for the next generation of clinical multimodality imaging applications. J Nucl Med : Off Publ, Soc Nucl Med.

[4] Gutberlet M, Mehl S, Frohlich M, Hausmann H, Plotkin M, Ruf J (2006). Determination of ventricular volumes in coronary artery disease: comparison of two gated SPECT analysis tools with MRI. Nuklearmedizin Nucl Med.

[5] von Knobelsdorff-Brenkenhoff F, Schulz-Menger J (2015) Role of cardiovascular magnetic resonance in the guidelines of the European Society of Cardiology. J Cardiovasc Magnet Reson 18(1) 10.1186/s12968-016-0225-6 PMC4724113 26800662

[6] Adenaw N, Salerno M (2013) PET/MRI: Current state of the art and future potential for cardiovascular applications. J Nuclear Cardiol : Off Publ Am Soc Nuclear Cardiol 10.1007/s12350-013-9780-5 23996656

[7] Bratis K, Mahmoud I, Chiribiri A, Nagel E (2013). Quantitative myocardial perfusion imaging by cardiovascular magnetic resonance and positron emission tomography. J Nucl Cardiol : Off Publ Am Soc Nucl Cardiol.

[8] Nekolla SG, Martinez-Moeller A, Saraste A (2009). PET and MRI in cardiac imaging: from validation studies to integrated applications. Eur J Nucl Med Mol Imaging.

[9] Nensa F, Tezgah E, Poeppel TD, Jensen CJ, Schelhorn J, Kohler J (2015). Integrated 18F-FDG PET/MR imaging in the assessment of cardiac masses: a pilot study. J Nucl Med : Off Publ, Soc Nucl Med.

[10] Rischpler C, Nekolla SG, Kunze KP, Schwaiger M (2015). PET/MRI of the heart. Semin Nucl Med.

[11] Nensa F, Poeppel TD, Beiderwellen K, Schelhorn J, Mahabadi AA, Erbel R (2013). Hybrid PET/MR imaging of the heart: feasibility and initial results. Radiology.

[12] Slart RH, Bax JJ, de Jong RM, de Boer J, Lamb HJ, Mook PH (2004). Comparison of gated PET with MRI for evaluation of left ventricular function in patients with coronary artery disease. J Nucl Med : Off Publ, Soc Nucl Med.

[13] Khorsand A, Graf S, Frank H, Kletter K, Sochor H, Maurer G (2003). Model-based analysis of electrocardiography-gated cardiac 18F-FDG PET images to assess left ventricular geometry and contractile function. J Nucl Med.

[14] Schaefer WM, Lipke CS, Nowak B, Kaiser HJ, Buecker A, Krombach GA (2003). Validation of an evaluation routine for left ventricular volumes, ejection fraction and wall motion from gated cardiac FDG PET: a comparison with cardiac magnetic resonance imaging. Eur J Nucl Med Mol Imaging.

[15] White HD, Norris RM, Brown MA, Brandt PW, Whitlock RM, Wild CJ (1987). Left ventricular end-systolic volume as the major determinant of survival after recovery from myocardial infarction. Circulation.

[16] Scholtens AM, Verberne HJ, Budde RP, Lam MG (2016). Additional heparin preadministration improves cardiac glucose metabolism suppression over low-carbohydrate diet alone in (1)(8)F-FDG PET imaging. J Nucl Med : Off Publ, Soc Nucl Med.

[17] Boellaard R, Delgado-Bolton R, Oyen WJ, Giammarile F, Tatsch K, Eschner W (2015). FDG PET/CT: EANM procedure guidelines for tumour imaging: version 2.0. Eur J Nucl Med Mol Imaging.

[18] Kobylecka M, Plazinska MT, Mazurek T, Bajera A, Slowikowska A, Fronczewska-Wieniawska K (2015). Simplified protocol of cardiac 18F-fluorodeoxyglucose positron emission tomography viability study in normoglycemic patients with known coronary artery disease. Clin Imaging.

[19] Vitale GD, de Kemp RA, Ruddy TD, Williams K, Beanlands RS (2001). Myocardial glucose utilization and optimization of (18)F-FDG PET imaging in patients with non-insulin-dependent diabetes mellitus, coronary artery disease, and left ventricular dysfunction. J Nucl Med : Off Publ, Soc Nucl Med.

[20] Eiber M, Martinez-Moller A, Souvatzoglou M, Holzapfel K, Pickhard A, Loffelbein D (2011). Value of a Dixon-based MR/PET attenuation correction sequence for the localization and evaluation of PET-positive lesions. Eur J Nucl Med Mol Imaging.

[21] Sattler B, Jochimsen T, Barthel H, Sommerfeld K, Stumpp P, Hoffmann KT (2013). Physical and organizational provision for installation, regulatory requirements and implementation of a simultaneous hybrid PET/MR-imaging system in an integrated research and clinical setting. MAGMA.

[22] AlJaroudi W, Jaber WA, Grimm RA, Marwick T, Cerqueira MD (2012). Alternative methods for the assessment of mechanical dyssynchrony using phase analysis of gated single photon emission computed tomography myocardial perfusion imaging. Int J Cardiovasc Imag.

[23] Ficaro EP, Lee BC, Kritzman JN, Corbett JR (2007). Corridor4DM: the Michigan method for quantitative nuclear cardiology. J Nucl Cardiol : Off Publ Am Soc Nucl Cardiol.

[24] Altman DG, Bland JM (1986). Comparison of methods of measuring blood pressure. J Epidemiol Community Health.

[25] Thormer G, Bertram HH, Garnov N, Peter V, Schutz T, Shang E (2013). Software for automated MRI-based quantification of abdominal fat and preliminary evaluation in morbidly obese patients. J Magnet Reson Imag : JMRI.

[26] Beerbaum P, Barth P, Kropf S, Sarikouch S, Kelter-Kloepping A, Franke D (2009). Cardiac function by MRI in congenital heart disease: impact of consensus training on interinstitutional variance. J Magnet Reson Imag : JMRI.

[27] Martinez-Moller A, Zikic D, Botnar RM, Bundschuh RA, Howe W, Ziegler SI (2007). Dual cardiac-respiratory gated PET: implementation and results from a feasibility study. Eur J Nucl Med Mol Imaging.

[28] Kumita S, Cho K, Nakajo H, Toba M, Uwamori M, Mizumura S (2001). Assessment of left ventricular diastolic function with electrocardiography-gated myocardial perfusion SPECT: comparison with multigated equilibrium radionuclide angiography. J Nucl Cardiol : Off Publ Am Soc Nucl Cardiol.

[29] Ioannidis JP, Trikalinos TA, Danias PG (2002). Electrocardiogram-gated single-photon emission computed tomography versus cardiac magnetic resonance imaging for the assessment of left ventricular volumes and ejection fraction: a meta-analysis. J Am Coll Cardiol.

[30] de Groot M, Meeuwis APW, Kok PJM, Corstens FHM, Oyen WJG (2005). Influence of blood glucose level, age and fasting period on non-pathological FDG uptake in heart and gut. Eur J Nucl Med Mol Imaging.

[31] Williams G, Kolodny GM (2008). Suppression of myocardial 18F-FDG uptake by preparing patients with a high-fat, low-carbohydrate diet. Am J Roentgenol.

[32] Slavin GS, Bluemke DA (2005). Spatial and temporal resolution in cardiovascular MR imaging: review and recommendations. Radiology.

[33] Oehmigen M, Ziegler S, Jakoby BW, Georgi JC, Paulus DH, Quick HH (2014). Radiotracer dose reduction in integrated PET/MR: implications from national electrical manufacturers association phantom studies. J Nucl Med : Off Publ, Soc Nucl Med.

[34] Nensa F, Tezgah E, Schweins K, Goebel J, Heusch P, Nassenstein K et al. (2016) Evaluation of a low-carbohydrate diet-based preparation protocol without fasting for cardiac PET/MR imaging. J Nucl Cardiol : Off Publ Am Soc Nucl Cardiol 10.1007/s12350-016-0443-1 26993494

[35] Salomon A, Goedicke A, Schweizer B, Aach T, Schulz V (2011). Simultaneous reconstruction of activity and attenuation for PET/MR. IEEE Trans Med Imaging.

